# Factors Influencing Emergence of Tularemia, Hungary, 1984–2010

**DOI:** 10.3201/eid1808.111826

**Published:** 2012-08

**Authors:** Miklós Gyuranecz, Jenő Reiczigel, Katalin Krisztalovics, László Monse, Gabriella Kükedi Szabóné, Andrásné Szilágyi, Bálint Szépe, László Makrai, Tibor Magyar, Mangesh Bhide, Károly Erdélyi

**Affiliations:** Center for Agricultural Research– Hungarian Academy of Sciences, Budapest, Hungary (M. Gyuranecz, T. Magyar);; University of Veterinary Medicine and Pharmacy, Košice, Slovakia (M. Gyuranecz, M. Bhide);; Slovak Academy of Sciences, Košice (M. Gyuranecz, M. Bhide);; Szent István University, Budapest (J. Reiczigel, L. Makrai);; National Center for Epidemiology, Budapest (K. Krisztalovics, A. Szilágyi);; Euroharex Ltd., Szolnok, Hungary (L. Monse);; Central Agriculture Office, Budapest (G. Kükedi Szabóné, K. Erdélyi);; and Medo Ltd., Tiszanána, Hungary (B. Szépe); and Institute of Neuroimmunology, Bratislava, Slovakia (M. Bhide)

**Keywords:** tularemia, emergence, Hungary, central Europe, Francisella tularensis, bacteria, zoonoses, Francisella tularensis subsp. holarctica

**To the Editor:** *Francisella tularensis,* the etiologic agent of tularemia, is a highly infectious zoonotic agent. *F. tularensis* subsp. *holarctica* (type B) is found throughout the Northern Hemisphere and is the only subspecies found in Europe (*1*). Lagomorphs and rodents probably serve as the primary mammalian reservoir hosts, and hematophagous arthropods, such as ticks, play a role as vectors and hosts (*2**,**3*). Although *F. tularensis* is a potential agent of biological warfare and several emergences and reemergences of tularemia have been reported around the world (*1**,**4*), the epizootiology of the disease is only partially understood. The aim of our study was to analyze factors that influence the emergence of tularemia in Hungary.

The study area (15,475 km^2^) included 3 counties in eastern Hungary. The analyzed data represented a period of 25 years, March 1984–February 2010. Annual *F. tularensis*–specific seroprevalence data for the European brown hare (*Lepus europaeus*) population were obtained by slide agglutination testing during the winter (December and January) screening of 2,500–25,000 animals (Technical Appendix). Population density data (animals/km^2^) for hares were based on February line transect counts and were obtained from the Hungarian Game Management database (www.vvt.gau.hu/vadgazdalkodasi_statisztikak.htm). Common vole (*Microtus arvalis*) densities (calculated from the number of active burrows/hectare during November) for 1996–2010 were obtained from the Central Agriculture Office, Budapest, Hungary. Vole density was scaled from 0 (absent) to 10 (peak population). The annual number of tularemia cases in humans (based on clinical history and tube agglutination test results) was obtained from the National Center for Epidemiology, Budapest.

The data were regrouped according to the yearly biologic cycle (March–February) for hares and voles (Figure), and relationships between these yearly data were quantified by using the Spearman rank correlation coefficient (*5*) at county and regional levels. A 2–3 year cycle was characteristic for the analyzed data. A significant positive correlation was found among the number of tularemia cases in humans and the seroprevalence of *F. tularensis* among European brown hares (Spearman ρ = 0.73; p<0.0001) and the population density of common voles (Spearman ρ = 0.77; p = 0.0081). A significant negative correlation was found between the population density of hares and the seroprevalence of *F. tularensis* in hares (Spearman ρ = −0.41; p = 0.0365).

**Figure:**
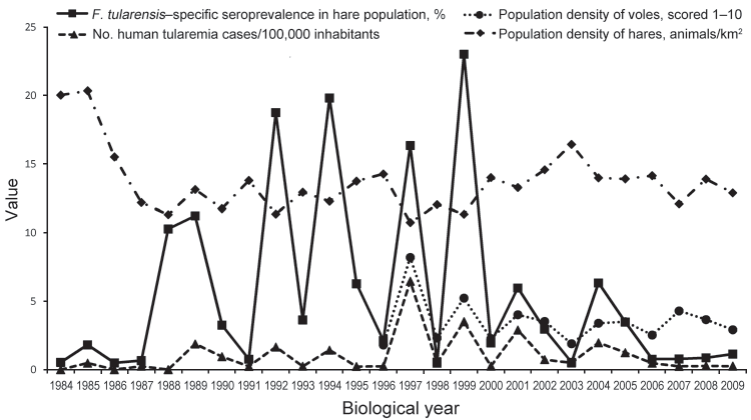
Correlation between the seroprevalence of *Francisella tularensis* in the European brown hare (*Lepus europaeus*) population, the population density of European brown hares and common voles (*Microtus arvalis*), and the number of tularemia cases in humans eastern Hungary, 1984–2010. Values were determined on the basis of biological years (March–February). Median values from the records of 3 counties were used for analysis.

The comprehensive and long-term annual data used in this study provide clues regarding the factors shaping the intraannual epizootiology and emergence or reemergence of tularemia. The European brown hare is moderately sensitive to *F. tularensis* subsp. *holarctica*. The hares produce a heterogeneous response to infection, which means that some die of overwhelming bacteremia and others survive with a protracted course of infection, thereby contributing to the maintenance of tularemia over longer periods and serving as useful sentinels of disease activity. Other studies have concluded that hares, together with infected ticks, may serve as disease reservoirs between epizootics (*2**,**3**,**6**,**7*).

However, we instead hypothesize that the 2–3 year cycling feature of the population dynamics for the common vole (*2*) determines the ecology of *F. tularensis* subsp. *holarctica* in eastern Hungary. The common vole is highly susceptible to *F. tularensis* subsp. *holarctica* (*3**,**8*). When population densities among voles are high, *F. tularensis* disease transmission and spillover to hares may be facilitated by stress-related aggression, cannibalism, and *F. tularensis* contamination of the environment by infectious body discharges (*2*). Enhanced transmission and spillover can expand local outbreaks to epizootic proportions, and infected hares may, in turn, further enhance the spread of disease through bacterial shedding in urine (*6**,**7*).

The disease in hares often results in septicemia and death (*7*), thus decreasing the population density of these animals. Hares and especially voles are also hosts for different stages of several tick species (*2**,**6*), so it can be expected, that higher numbers of infected rodents and lagomorphs result in an increased proportion of infected ticks and, thus, increased transmission of *F. tularensis* subsp. *holarctica*. It can be concluded that a higher number of infection sources in the environment results in elevated numbers of cases in humans, mainly through the handling and skinning of hares, but also through tick bites and, potentially, the inhalation of infectious aerosols originating from, for example, hay or grain.

Technical Appendix Detailed data of the annual *Francisella tularensis*–specific seroprevalence in the European brown hare (*Lepus europaeus*) population, the annual population density of European brown hares and common voles (*Microtus arvalis*), and the annual number of tularemia cases in humans, Hungary, 1984–2010. Data are for 3 counties in eastern Hungary: Békés, Csongrád, and Jász-Nagykun-Szolnok)*.

## References

[1] Petersen JM, Schriefer ME. Tularemia: emergence/re-emergence. Vet Res. 2005;36:455–67. 10.1051/vetres:2005006 15845234

[2] Friend M. Tularemia, 1st ed. Reston (VA): US Geological Survey, circular 1297; 2006 [cited 2011 Feb 4]. http://www.nwhc.usgs.gov/publications/tularemia

[3] Mörner T, Addison E. Tularemia. In: Williams ES, Barker IK, editors. Infectious diseases of wild mammals, 3rd ed. Ames (IA): Iowa University Press; 2001. p. 303–12.

[4] Kaysser P, Seibold E, Matz-Rensing K, Pfeffer M, Essbauer S, Splettstoesser WD. Re-emergence of tularemia in Germany: presence of *Francisella tularensis* in different rodent species in endemic areas. BMC Infect Dis. 2008;8:157. 10.1186/1471-2334-8-157 19014635 PMC2629769

[5] R program. R Foundation for Statistical Computing, version 2.13.1. 2011 [cited 2011 Feb 4]. http://ftp5.gwdg.de/pub/misc/cran

[6] Gyuranecz M, Rigó K, Dán Á, Földvári G, Makrai L, Dénes B, Investigation of the ecology of *Francisella tularensis* during an inter-epizootic period. Vector Borne Zoonotic Dis. 2011;11:1031–5. 10.1089/vbz.2010.0091 21142970

[7] Gyuranecz M, Szeredi L, Makrai L, Fodor L, Ráczné Mészáros Á, Szépe B, Tularemia of European brown hare (*Lepus europaeus*): a pathological, histopathological and immunhistochemical study. Vet Pathol. 2010;47:958–63. 10.1177/0300985810369902 20466857

[8] World Health Organization. WHO guidelines on tularemia. 2007 [cited 2011 Feb 4]. http://www.cdc.gov/tularemia/resources/whotularemiamanual.pdf

